# Ketamine: A Potential Adjunct for Severe Benzodiazepine Withdrawal

**DOI:** 10.7759/cureus.20114

**Published:** 2021-12-02

**Authors:** Kristen Purcell, Pollianne W Bianchi, Daniel Glenn, Brandon Blakey, Sergey Motov

**Affiliations:** 1 Emergency Medicine, Midwestern University Chicago College of Osteopathic Medicine, Downers Grove, USA; 2 Emergency Medicine, Crozer-Keystone Health System, Upland, USA; 3 Medicine, Philadelphia College of Osteopathic Medicine, Philadelphia, USA; 4 Emergency Medicine, Crozer-Keystone Health System, Chester, USA; 5 Emergency Medicine, Maimonides Medical Center, Brooklyn, USA

**Keywords:** emergency medicine, toxicology, ketamine, withdrawal, substance abuse, benzodiazepine

## Abstract

Following the abrupt cessation of benzodiazepine therapy, patients can present with acute life-threatening withdrawal. Medical management of benzodiazepine withdrawal is typically undertaken with benzodiazepines either through loading dose with gradual taper or symptom triggered treatment, though adjuvant anxiolytics and anticonvulsants are often used. Ketamine, increasingly utilized as an adjunct in the treatment of alcohol withdrawal, may represent an effective medication in the treatment of benzodiazepine withdrawal. In this case report, a 27-year-old male with a history of benzodiazepine and opioid abuse presented to our emergency department with a chief complaint of drug withdrawal. Despite standard treatment with large amounts of benzodiazepine, barbiturate, opioid, and adjunctive medications, the patient remained with severe withdrawal syndrome until an infusion of ketamine (0.5mg/kg in 30 minutes) was administered resulting in significant improvement of the patient symptoms. This case demonstrates the potential role of ketamine as an adjunct medication in the treatment of benzodiazepine withdrawal.

## Introduction

Benzodiazepines (BZDs) induce allosteric modulation of the γ-aminobutyric acid (GABA)-A channel, allowing for an enhanced influx of chloride following GABA activation of its respective receptor, resulting in hyperpolarization of neurons [1]. These medications have a broad clinical range as anxiolytics, muscle relaxers, anticonvulsants, and sedatives but are most commonly prescribed for anxiety and insomnia. Following the abrupt cessation of benzodiazepine therapy, patients can present with an acute withdrawal syndrome which may include symptoms such as anxiety, tremors, insomnia, agitation, hypertension, tachycardia, altered sensation, diaphoresis, and seizures [2]. Medical management of life-threatening withdrawal is typically undertaken with either benzodiazepine loading and gradual taper or symptom-triggered treatment, though adjuvant anxiolytics and anticonvulsants are often used [3,4]. Severe withdrawal symptoms may require high benzodiazepine doses, placing patients at risk for respiratory depression. In these cases, ketamine may be a beneficial adjunct treatment for benzodiazepine withdrawal as it has been shown to provide γ-aminobutyric acid receptor agonism, decrease glutamate activity, has a low potential for respiratory depression, and has been used as an effective adjunctive treatment in severe alcohol withdrawal [5,6]. Currently, ketamine is FDA-approved for anesthesia and sedation but has off-label indications for chronic pain management and post-traumatic stress disorder (PTSD). This case report presents another possible off-label use of ketamine in the management of benzodiazepine withdrawal.

## Case presentation

A 27-year-old male with a history of benzodiazepine and opioid abuse presented to our emergency department with a chief complaint of drug withdrawal. The patient noted significant polysubstance use, including 30mg of oral alprazolam and 10 bags of insufflated fentanyl consumption daily. On arrival, the patient was found to manifest symptoms consistent with a combined opioid and benzodiazepine withdrawal, including palpitations, tremors, agitation, nausea, and diarrhea. His physical examination was consistent with withdrawal including mild tachycardia, resting tremor, and mydriasis. Attempted symptomatic control was initiated with lorazepam 4mg. In addition, buprenorphine therapy was initiated with a starting dose of 4mg. Despite these measures, the patient continued to remain symptomatic with agitation, tremors, and nausea, requiring increasing doses of both benzodiazepines and buprenorphine. He received an additional 12mg of buprenorphine in addition to 45mg of diazepam. A multimodal approach to symptom control was attempted involving olanzapine 10mg, gabapentin 300mg, phenobarbital 260mg, and ondansetron 4mg. At this point in treatment, the patient’s opioid withdrawal symptoms appeared to be under control with buprenorphine therapy, however, his benzodiazepine withdrawal symptoms of tremor, agitation tachycardia, and hypertension continued to predominate, requiring repeated dosing of benzodiazepines, escalating to an additional 60mg of diazepam (for a total of 105mg of diazepam). 

Due to concerns of respiratory depression in the setting of escalating benzodiazepine dosages with buprenorphine combination therapy and in an attempt to better control the patient’s withdrawal symptoms the patient was placed on a ketamine infusion of 0.5mg/kg given over a 30-minute interval. The patient had a profound response to ketamine both subjectively and objectively by improvement of his tremor and agitation as well as resolution of tachycardia and hypertension. The patient was admitted to the intensive care unit for further treatment and monitoring, placed on a benzodiazepine taper protocol by the inpatient team, and several days later was ultimately discharged successfully without the need for further escalation of therapy.

## Discussion

Since 2003, ambulatory care visits related to BZDs doubled, and in 2008, 5.2% of the population in the United States filled one or more BZD prescriptions [7,8]. The number of BZD-related visits increased not only for new prescribers but predominantly for those receiving BZDs on a long-term basis. The growing number of BZD prescriptions is a concern given the abuse potential and risks associated with BZD use [9]. The danger of BZD overdose is highest when these medications are used in conjunction with opioids or alcohol, however, death resulting from BZD withdrawal has also been described [10,11]. Withdrawal symptoms can include irritability, insomnia, panic attacks, concentration impairment, tachycardia, hypertension, and muscle spasms with more severe symptoms such as seizures following withdrawal from long-term use at higher doses. Populations with the highest risk for BZD withdrawal are those who are prescribed for prolonged periods of time (>4 weeks) or short-acting doses. Sensitivity to BZDs can start to decline in just over a week, indicating possible dependence and therefore withdrawal risk [12].

The current standard of treatment for BZD withdrawal is the administration of long-acting BZDs. While potential adjunct or stand-alone therapies have been explored such as beta-blockers (propranolol), GABA-B agonists (baclofen), N-methyl-D-aspartate (NMDA) antagonists (lamotrigine), and neuroleptic (trazodone), there remains a lack of data for these therapies as a first-line treatment in BZD withdrawal [13]. As the number of BZD prescriptions increases, there is an emerging concern for treating withdrawal syndromes in patients, particularly in those who have a high tolerance following long-term BZD use, as these patients often require high doses of BZDs for symptomatic control. Classically, higher BZD dosages have prompted concern for the potential of the critical central nervous system (CNS) depression and respiratory compromise especially in the setting of combined use with narcotics and alcohol, though no high-quality literature has consistently shown benzodiazepine exclusive respiratory depression to be a significant risk. Nonetheless, ketamine may represent a novel approach for supplemental BZD withdrawal therapy.

Ketamine is a phencyclidine analog that was initially recognized for its anesthetic properties and has been more recently evaluated for its use as an analgesic, antidepressant, and anxiolytic [14]. Ketamine use has become increasingly popular for nonopioid pain control, sedation of agitated patients, and procedural analgesia and anesthesia [15,16]. While undesired side effects of subanesthetic ketamine such as nausea, vomiting, and hallucinations remain a concern, ketamine is considered generally safe with a wide therapeutic window [14].

Ketamine’s mechanisms of action include NMDA receptor antagonism and agonism of the GABA-A as well as GABA-B receptor [17]. NMDA receptor antagonism has shown to be effective in alleviating BZD withdrawal symptoms in the mouse model and GABA-B agonists have shown similar responses in clinical trials, though ketamine itself has not been adequately described in the literature as a potential treatment for BZD withdrawal in human cases [13,18,19]. A previous case report noted that low-dose ketamine administration in a chronically ill patient with opioid and BZD dependence decreased medication requirements for the management of their delirium and agitation during admission [20].

The case is notably limited by a number of potential confounders. Due to the polysubstance nature of the patient’s withdrawal syndrome, an isolated BZD effect is difficult to conclude, as there is often substantial overlap between opioid and benzodiazepine withdrawal. Additionally, the patient may likely have benefited from additional BZD dosing. Finally, no conclusion can be made whether the patient’s improvement was a function of the previous medications administered or an effect driven by ketamine administration. Nonetheless, the case presented demonstrates the potential role of ketamine as an adjunct medication in the treatment of BZD withdrawal.

## Conclusions

As BZD prescriptions increase, there is a concern for patients presenting to the emergency department for symptoms related to BZD withdrawal. Current standard treatment of BZD withdrawal includes long-acting BZDs while adjunctive medications can be administered for additional support of withdrawal symptom management. Ketamine demonstrates antagonism of the NMDA receptor as well as agonism of the GABA-A and GABA-B receptors making it an intuitive potential adjunctive medication for BZD withdrawal. In this case study, the patient presented with combined BZD and opioid withdrawal requiring increasing amounts of BZDs for symptomatic control. Given the concern of respiratory depression with coadministration of BZDs and buprenorphine, a subanesthetic dose of ketamine was administered as an adjunctive medication resulting in significant improvement of the patient's withdrawal symptoms. In future research, patient populations with isolated BZD withdrawal should be evaluated for symptom response to subanesthetic doses of ketamine as ketamine may represent an effective adjunct medication in the treatment of BZD withdrawal.
